# (*S_p_*)-1-Diphenyl­phosphanyl-2-{(*S*)-[2-(diphenyl­phosphan­yl)phen­yl]hydroxy­meth­yl}ferrocene

**DOI:** 10.1107/S1600536808038294

**Published:** 2008-11-20

**Authors:** Jan W. Bats, Angelino Doppiu, Andreas Rivas Nass, A. Stephen K. Hashmi

**Affiliations:** a Institut für Organische Chemie, Universität Frankfurt, Max-von-Laue-Strasse 7, D-60438 Frankfurt am Main, Germany; b Umicore AG & Co. KG, Strategic Research and Development, Precious Metals Chemistry, Rodenbacher Chaussee 4, D-63457 Hanau, Germany; c Organisch-Chemisches Institut, Universität Heidelberg, Im Neuenheimer Feld 270, D-69120 Heidelberg, Germany

## Abstract

The absolute configuration of the title compound, [Fe(C_5_H_5_)(C_36_H_29_OP_2_)], is *S_p_* at the ferrocene group and *S* at the asymmetric C atom. Both P atoms have a trigonal–pyramidal conformation. There is a short intra­molecular C—H⋯P contact with an H⋯P distance of 2.56 Å. The hydr­oxy group is involved in an intra­molecular O—H⋯π_phen­yl_ inter­action. The crystal packing shows five very weak inter­molecular C—H⋯π contacts, with H⋯*Cg* distances between 3.26 and 3.39 Å (*Cg* is the centroid of a phenyl or cyclo­penta­dienyl ring).

## Related literature

The preparation of the title compound has been reported by Lotz & Spindler (2005 ▶). The stereochemistry of the Taniaphos ligand has been discussed by Ireland *et al.* (2008 ▶). For the synthesis of related compounds, see: Ireland *et al.* (2002 ▶); Fukuzawa, Yamamoto, Hosaka & Kikuchi (2007 ▶). For the crystal structures of related compounds, see: Fukuzawa, Yamamoto & Kikuchi (2007 ▶); Ireland *et al.* (1999 ▶).
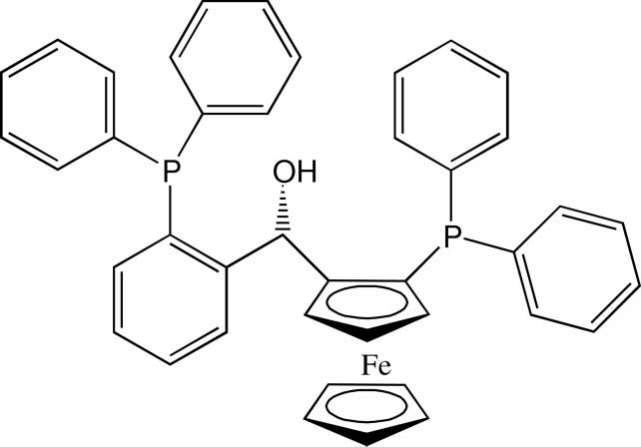


## Experimental

### 

#### Crystal data

[Fe(C_5_H_5_)(C_36_H_29_OP_2_)]
*M* *_r_* = 660.47
Monoclinic, 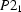

*a* = 11.6111 (15) Å
*b* = 8.6154 (10) Å
*c* = 16.481 (2) Å
β = 97.807 (12)°
*V* = 1633.4 (3) Å^3^
*Z* = 2
Mo *K*α radiation
μ = 0.59 mm^−1^
*T* = 162 (2) K
0.40 × 0.40 × 0.32 mm

#### Data collection

Siemens SMART 1K CCD diffractometer
Absorption correction: numerical (*SHELXTL*; Sheldrick, 2008 ▶) *T*_min_ = 0.795, *T*_max_ = 0.845
25293 measured reflections
9131 independent reflections
7926 reflections with *I* > 2σ(*I*)
*R*_int_ = 0.051

#### Refinement

*R*[*F* ^2^ > 2σ(*F* ^2^)] = 0.038
*wR*(*F* ^2^) = 0.083
*S* = 1.07
9131 reflections
410 parameters
1 restraint
H atoms treated by a mixture of independent and constrained refinement
Δρ_max_ = 0.33 e Å^−3^
Δρ_min_ = −0.23 e Å^−3^
Absolute structure: Flack (1983 ▶), 4069 Friedel pairs
Flack parameter: −0.023 (10)

### 

Data collection: *SMART* (Siemens, 1995 ▶); cell refinement: *SMART*; data reduction: *SAINT* (Siemens, 1995 ▶); program(s) used to solve structure: *SHELXS97* (Sheldrick, 2008 ▶); program(s) used to refine structure: *SHELXL97* (Sheldrick, 2008 ▶); molecular graphics: *SHELXTL* (Sheldrick, 2008 ▶); software used to prepare material for publication: *SHELXL97*.

## Supplementary Material

Crystal structure: contains datablocks global, I. DOI: 10.1107/S1600536808038294/nc2123sup1.cif

Structure factors: contains datablocks I. DOI: 10.1107/S1600536808038294/nc2123Isup2.hkl

Additional supplementary materials:  crystallographic information; 3D view; checkCIF report

## Figures and Tables

**Table 1:** Hydrogen-bond geometry (Å, °)

*D*—H⋯*A*	*D*—H	H⋯*A*	*D*⋯*A*	*D*—H⋯*A*
C11—H11*A*⋯P1	1.00	2.56	3.153 (2)	118
O1—H1*A*⋯C23	0.78 (3)	2.51 (3)	3.217 (3)	152 (2)

**Fig. 1.**
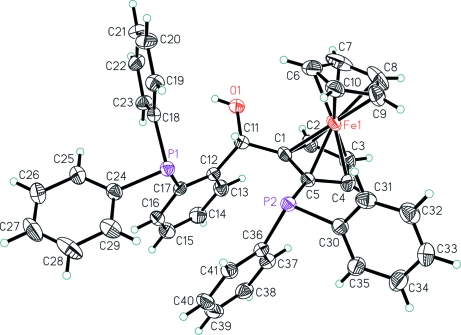
The structure of the title compound shown with 50% probability displacement ellipsoids. The H atoms are drawn as small spheres of arbitrary radius.

## supplementary crystallographic information

### Comment

The structure originally published for the Taniaphos ligands, a chiral ligand technology owned by Umicore and sold *via* Solvias, recently had to be corrected. It was shown that these ligands do not have the (*R*,*S*_p_) or (S,*R*_p_) but the (*R*,*R*_p_) or (S,*S*_p_) configuration, respectively (Fukuzawa, Yamamoto, Hosaka & Kikuchi, *et al.*, 2007; Ireland *et al.*,  2008). The planar chirality of the 1,2-disubstituted ferrocene initially had been assigned incorrectly. Our present investigation confirms the configuration of a special member of this family, bearing a hydroxy group in the side-chain of the ferrocene ring (Lotz & Spindler, 2005).

The molecular structure of the title compound is shown in Fig. 1. The ferrocene group deviates only 4° from an eclipsed conformation. The angle between the planes of the two cyclopentadienyl rings is 4.2 (2)°. Both P atoms have a pyramidal conformation. The lone-pair lobe of atom P1 shows a short intramolecular contact distance of 2.56 Å with the H atom of C11 (Table 1). The hydroxy group is not involved in conventional inter- or intramolecular hydrogen bonding. It shows instead a short intramolecular O—H···π_phenyl_ interaction with the phenyl ring labeled C18 through C23. The O—H group is not directed to the center of this phenyl ring, but points mainly to atom C23 (Table 1). There is an intramolecular π···π interaction between the benzene rings labeled C12 → C17 and C36 → C41. The angle between the planes of these rings is 4.8 (2)°. The shortest contact distance is 3.498 (3)Å between C12 and C37. The crystal packing shows five very weak intermolecular C—H···π interactions, with H···*Cg* distances between 3.26 and 3.39 Å (*Cg* is the centroid of a phenyl or cyclopentadienyl ring).

### Experimental

The preparation of the title compound has been reported by Lotz & Spindler (2005). Crystals were obtained from a solution of the title compound in a mixture of chloroform and n-hexane.

### Refinement

H atoms were geometrically positioned using distances: C_planar_—H=0.95 Å, C_primary_—H=1.00 Å, *U*_iso_(H)=1.2*U*_eq_(C). The H atom of the hydroxy group was taken from a difference Fourier synthesis and was refined with an isotropic thermal parameter. Friedel opposites were not averaged. The absolute configuration was determined from 4069 Friedel pairs.

### Crystal data

**Table:**

[Fe(C_5_H_5_)(C_36_H_29_OP_2_)]	*F*_000_ = 688
*M**_r_* = 660.47	*D*_x_ = 1.343 Mg m^−^^3^
Monoclinic, *P*2_1_	Mo *K*α radiation λ = 0.71073 Å
Hall symbol: P 2yb	Cell parameters from 239 reflections
*a* = 11.6111 (15) Å	θ = 3–23º
*b* = 8.6154 (10) Å	µ = 0.59 mm^−^^1^
*c* = 16.481 (2) Å	*T* = 162 (2) K
β = 97.807 (12)º	Block, orange
*V* = 1633.4 (3) Å^3^	0.40 × 0.40 × 0.32 mm
*Z* = 2	

### Data collection

**Table:**

Siemens SMART 1K CCD diffractometer	9131 independent reflections
Radiation source: normal-focus sealed tube	7926 reflections with *I* > 2σ(*I*)
Monochromator: graphite	*R*_int_ = 0.051
*T* = 162(2) K	θ_max_ = 30.5º
ω scans	θ_min_ = 1.8º
Absorption correction: numerical(SHELXTL; Sheldrick, 2008)	*h* = −16→16
*T*_min_ = 0.795, *T*_max_ = 0.845	*k* = −12→12
25293 measured reflections	*l* = −23→23

### Refinement

**Table:**

Refinement on *F*^2^	Hydrogen site location: inferred from neighbouring sites
Least-squares matrix: full	H atoms treated by a mixture of independent and constrained refinement
*R*[*F*^2^ > 2σ(*F*^2^)] = 0.038	*w* = 1/[σ^2^(*F*_o_^2^) + (0.03*P*)^2^ + 0.5*P*] where *P* = (*F*_o_^2^ + 2*F*_c_^2^)/3
*wR*(*F*^2^) = 0.083	(Δ/σ)_max_ = 0.003
*S* = 1.07	Δρ_max_ = 0.33 e Å^−^^3^
9131 reflections	Δρ_min_ = −0.23 e Å^−^^3^
410 parameters	Extinction correction: none
1 restraint	Absolute structure: Flack (1983), 4069 Friedel pairs
Primary atom site location: structure-invariant direct methods	Flack parameter: −0.023 (10)
Secondary atom site location: difference Fourier map	

### Special details

**Table:**

Geometry. All e.s.d.'s (except the e.s.d. in the dihedral angle between two l.s. planes) are estimated using the full covariance matrix. The cell e.s.d.'s are taken into account individually in the estimation of e.s.d.'s in distances, angles and torsion angles; correlations between e.s.d.'s in cell parameters are only used when they are defined by crystal symmetry. An approximate (isotropic) treatment of cell e.s.d.'s is used for estimating e.s.d.'s involving l.s. planes.
Refinement. Refinement of *F*^2^ against ALL reflections. The weighted *R*-factor *wR* and goodness of fit *S* are based on *F*^2^, conventional *R*-factors *R* are based on *F*, with *F* set to zero for negative *F*^2^. The threshold expression of *F*^2^ > σ(*F*^2^) is used only for calculating *R*-factors(gt) *etc*. and is not relevant to the choice of reflections for refinement. *R*-factors based on *F*^2^ are statistically about twice as large as those based on *F*, and *R*- factors based on ALL data will be even larger.

### Fractional atomic coordinates and isotropic or equivalent isotropic displacement parameters (Å^2^)

**Table:**

	*x*	*y*	*z*	*U*_iso_*/*U*_eq_	
Fe1	0.19359 (2)	−0.01898 (3)	0.741420 (18)	0.02801 (7)	
P1	0.62868 (4)	0.27356 (6)	0.85276 (3)	0.02329 (11)	
P2	0.32422 (4)	0.33294 (6)	0.71106 (3)	0.02413 (11)	
O1	0.47730 (15)	−0.14478 (19)	0.81532 (11)	0.0352 (4)	
C1	0.35814 (16)	−0.0009 (2)	0.71422 (12)	0.0249 (4)	
C2	0.30376 (19)	−0.1365 (3)	0.67651 (15)	0.0319 (5)	
H2A	0.3304	−0.2402	0.6853	0.038*	
C3	0.2027 (2)	−0.0890 (3)	0.62352 (16)	0.0360 (5)	
H3A	0.1499	−0.1559	0.5913	0.043*	
C4	0.19426 (19)	0.0751 (3)	0.62691 (14)	0.0310 (5)	
H4A	0.1349	0.1367	0.5973	0.037*	
C5	0.29067 (17)	0.1323 (2)	0.68279 (12)	0.0244 (4)	
C6	0.2085 (2)	−0.0518 (3)	0.86549 (16)	0.0466 (7)	
H6A	0.2790	−0.0574	0.9021	0.056*	
C7	0.1497 (3)	−0.1778 (4)	0.8229 (2)	0.0585 (9)	
H7A	0.1742	−0.2831	0.8260	0.070*	
C8	0.0498 (3)	−0.1210 (4)	0.7757 (2)	0.0567 (8)	
H8A	−0.0053	−0.1812	0.7413	0.068*	
C9	0.0444 (2)	0.0418 (4)	0.78764 (17)	0.0445 (6)	
H9A	−0.0144	0.1098	0.7628	0.053*	
C10	0.1430 (2)	0.0839 (3)	0.84353 (14)	0.0365 (5)	
H10A	0.1619	0.1858	0.8630	0.044*	
C11	0.46677 (16)	0.0034 (2)	0.77503 (12)	0.0255 (4)	
H11A	0.4583	0.0857	0.8166	0.031*	
C12	0.57475 (16)	0.0380 (2)	0.73482 (12)	0.0232 (4)	
C13	0.59482 (18)	−0.0521 (2)	0.66787 (13)	0.0306 (5)	
H13A	0.5415	−0.1319	0.6489	0.037*	
C14	0.69159 (18)	−0.0268 (3)	0.62852 (13)	0.0345 (5)	
H14A	0.7051	−0.0905	0.5837	0.041*	
C15	0.76792 (19)	0.0911 (3)	0.65477 (14)	0.0341 (5)	
H15A	0.8330	0.1109	0.6271	0.041*	
C16	0.74938 (18)	0.1810 (3)	0.72182 (13)	0.0272 (4)	
H16A	0.8024	0.2621	0.7394	0.033*	
C17	0.65449 (17)	0.1546 (2)	0.76401 (12)	0.0224 (4)	
C18	0.64664 (17)	0.1267 (2)	0.93453 (12)	0.0247 (4)	
C19	0.59727 (19)	0.1590 (3)	1.00546 (13)	0.0309 (5)	
H19A	0.5589	0.2553	1.0102	0.037*	
C20	0.6035 (2)	0.0530 (3)	1.06872 (14)	0.0380 (5)	
H20A	0.5703	0.0774	1.1168	0.046*	
C21	0.6582 (2)	−0.0893 (3)	1.06242 (14)	0.0345 (5)	
H21A	0.6609	−0.1630	1.1055	0.041*	
C22	0.70842 (19)	−0.1230 (3)	0.99310 (13)	0.0312 (5)	
H22A	0.7463	−0.2198	0.9888	0.037*	
C23	0.70385 (16)	−0.0159 (3)	0.92957 (12)	0.0284 (4)	
H23A	0.7397	−0.0395	0.8825	0.034*	
C24	0.76505 (18)	0.3831 (3)	0.87543 (13)	0.0284 (4)	
C25	0.8662 (2)	0.3235 (3)	0.91878 (17)	0.0450 (6)	
H25A	0.8679	0.2187	0.9369	0.054*	
C26	0.9648 (2)	0.4150 (4)	0.93611 (19)	0.0574 (8)	
H26A	1.0332	0.3731	0.9663	0.069*	
C27	0.9636 (3)	0.5670 (4)	0.90944 (17)	0.0511 (8)	
H27A	1.0310	0.6298	0.9215	0.061*	
C28	0.8645 (3)	0.6274 (3)	0.86535 (19)	0.0492 (7)	
H28A	0.8642	0.7313	0.8460	0.059*	
C29	0.7647 (2)	0.5367 (3)	0.84906 (16)	0.0369 (5)	
H29A	0.6960	0.5798	0.8198	0.044*	
C30	0.19045 (17)	0.4249 (2)	0.65968 (13)	0.0268 (4)	
C31	0.0981 (2)	0.4392 (3)	0.70517 (14)	0.0334 (5)	
H31A	0.1085	0.4098	0.7612	0.040*	
C32	−0.00866 (19)	0.4957 (3)	0.66960 (15)	0.0399 (5)	
H32A	−0.0713	0.5027	0.7010	0.048*	
C33	−0.0244 (2)	0.5420 (3)	0.58854 (17)	0.0431 (6)	
H33A	−0.0975	0.5809	0.5642	0.052*	
C34	0.0673 (2)	0.5313 (4)	0.54313 (16)	0.0473 (7)	
H34A	0.0572	0.5635	0.4875	0.057*	
C35	0.17429 (18)	0.4734 (3)	0.57875 (13)	0.0367 (5)	
H35A	0.2369	0.4670	0.5473	0.044*	
C36	0.43404 (17)	0.3780 (2)	0.64374 (12)	0.0249 (4)	
C37	0.45399 (18)	0.2857 (3)	0.57774 (13)	0.0301 (4)	
H37A	0.4039	0.2006	0.5620	0.036*	
C38	0.54639 (19)	0.3165 (3)	0.53441 (14)	0.0348 (5)	
H38A	0.5595	0.2522	0.4898	0.042*	
C39	0.6192 (2)	0.4415 (3)	0.55664 (15)	0.0377 (6)	
H39A	0.6822	0.4630	0.5271	0.045*	
C40	0.6003 (2)	0.5345 (3)	0.62161 (17)	0.0378 (5)	
H40A	0.6501	0.6203	0.6365	0.045*	
C41	0.50852 (17)	0.5034 (2)	0.66566 (14)	0.0301 (4)	
H41A	0.4965	0.5675	0.7107	0.036*	
H1A	0.532 (2)	−0.146 (3)	0.8487 (17)	0.034 (7)*	

### Atomic displacement parameters (Å^2^)

**Table:**

	*U*^11^	*U*^22^	*U*^33^	*U*^12^	*U*^13^	*U*^23^
Fe1	0.02189 (13)	0.02740 (15)	0.03497 (16)	−0.00588 (13)	0.00474 (11)	0.00120 (13)
P1	0.0218 (2)	0.0242 (2)	0.0240 (2)	−0.00007 (19)	0.00343 (19)	−0.0001 (2)
P2	0.0264 (2)	0.0262 (3)	0.0196 (2)	−0.0037 (2)	0.00265 (18)	−0.0002 (2)
O1	0.0310 (8)	0.0341 (9)	0.0392 (9)	−0.0060 (7)	0.0002 (7)	0.0144 (7)
C1	0.0224 (8)	0.0251 (10)	0.0276 (9)	−0.0054 (8)	0.0049 (7)	−0.0012 (8)
C2	0.0293 (11)	0.0288 (11)	0.0380 (12)	−0.0044 (9)	0.0062 (9)	−0.0060 (9)
C3	0.0340 (12)	0.0360 (12)	0.0370 (13)	−0.0084 (10)	0.0006 (10)	−0.0089 (10)
C4	0.0283 (11)	0.0328 (12)	0.0300 (11)	−0.0047 (9)	−0.0024 (9)	−0.0023 (9)
C5	0.0230 (9)	0.0262 (10)	0.0241 (10)	−0.0055 (8)	0.0032 (7)	0.0005 (8)
C6	0.0403 (12)	0.063 (2)	0.0393 (13)	0.0053 (12)	0.0143 (10)	0.0198 (12)
C7	0.0666 (19)	0.0405 (15)	0.078 (2)	−0.0039 (14)	0.0434 (17)	0.0172 (15)
C8	0.0426 (15)	0.0584 (19)	0.074 (2)	−0.0285 (14)	0.0247 (14)	−0.0143 (16)
C9	0.0246 (11)	0.0599 (17)	0.0501 (15)	0.0002 (11)	0.0098 (10)	0.0011 (13)
C10	0.0364 (12)	0.0422 (14)	0.0328 (12)	−0.0073 (10)	0.0113 (10)	0.0005 (10)
C11	0.0260 (9)	0.0249 (11)	0.0262 (9)	−0.0024 (8)	0.0053 (7)	0.0023 (8)
C12	0.0219 (9)	0.0245 (9)	0.0230 (9)	0.0006 (7)	0.0022 (7)	0.0039 (7)
C13	0.0302 (10)	0.0309 (12)	0.0304 (11)	−0.0023 (8)	0.0031 (8)	−0.0042 (8)
C14	0.0369 (10)	0.0417 (12)	0.0267 (10)	0.0016 (11)	0.0103 (8)	−0.0050 (11)
C15	0.0276 (10)	0.0458 (13)	0.0311 (11)	0.0004 (9)	0.0120 (9)	0.0007 (10)
C16	0.0238 (10)	0.0305 (11)	0.0280 (10)	−0.0031 (8)	0.0058 (8)	0.0013 (9)
C17	0.0206 (9)	0.0258 (10)	0.0206 (9)	0.0028 (7)	0.0018 (7)	0.0031 (7)
C18	0.0213 (9)	0.0288 (10)	0.0233 (9)	−0.0035 (8)	0.0008 (7)	0.0009 (8)
C19	0.0348 (11)	0.0328 (11)	0.0265 (10)	0.0005 (9)	0.0092 (9)	−0.0043 (9)
C20	0.0468 (13)	0.0450 (13)	0.0246 (11)	0.0002 (11)	0.0140 (10)	−0.0016 (10)
C21	0.0383 (12)	0.0403 (13)	0.0248 (11)	−0.0008 (10)	0.0045 (9)	0.0090 (9)
C22	0.0314 (10)	0.0345 (11)	0.0269 (11)	0.0053 (9)	0.0008 (8)	0.0033 (8)
C23	0.0285 (9)	0.0334 (10)	0.0241 (9)	0.0056 (10)	0.0066 (7)	0.0033 (9)
C24	0.0297 (10)	0.0302 (11)	0.0257 (10)	−0.0069 (8)	0.0056 (8)	−0.0048 (8)
C25	0.0334 (12)	0.0499 (15)	0.0488 (15)	−0.0136 (12)	−0.0047 (10)	0.0138 (12)
C26	0.0378 (14)	0.083 (2)	0.0483 (16)	−0.0260 (15)	−0.0056 (12)	0.0108 (15)
C27	0.0493 (16)	0.0645 (19)	0.0433 (15)	−0.0342 (14)	0.0194 (13)	−0.0207 (14)
C28	0.0579 (17)	0.0324 (13)	0.0644 (18)	−0.0186 (12)	0.0343 (15)	−0.0121 (12)
C29	0.0401 (12)	0.0304 (11)	0.0434 (13)	−0.0024 (9)	0.0167 (10)	−0.0038 (10)
C30	0.0267 (9)	0.0261 (10)	0.0278 (10)	−0.0013 (8)	0.0041 (8)	−0.0010 (8)
C31	0.0383 (12)	0.0312 (12)	0.0330 (11)	0.0020 (9)	0.0130 (9)	0.0015 (9)
C32	0.0330 (11)	0.0404 (13)	0.0489 (14)	0.0020 (11)	0.0149 (10)	0.0006 (12)
C33	0.0271 (11)	0.0507 (15)	0.0502 (15)	0.0051 (10)	0.0009 (10)	0.0023 (12)
C34	0.0368 (13)	0.071 (2)	0.0337 (13)	0.0077 (12)	0.0017 (10)	0.0095 (12)
C35	0.0302 (10)	0.0532 (14)	0.0276 (10)	0.0050 (11)	0.0068 (8)	0.0037 (11)
C36	0.0249 (9)	0.0246 (10)	0.0247 (10)	0.0011 (8)	0.0016 (7)	0.0041 (8)
C37	0.0311 (10)	0.0330 (11)	0.0256 (10)	−0.0017 (9)	0.0018 (8)	0.0027 (9)
C38	0.0355 (11)	0.0439 (13)	0.0258 (10)	0.0052 (10)	0.0070 (9)	0.0047 (9)
C39	0.0322 (11)	0.0399 (14)	0.0432 (13)	0.0044 (9)	0.0136 (10)	0.0164 (10)
C40	0.0315 (11)	0.0270 (11)	0.0553 (15)	−0.0037 (9)	0.0073 (10)	0.0118 (10)
C41	0.0283 (9)	0.0239 (11)	0.0379 (11)	0.0009 (8)	0.0042 (8)	0.0041 (9)

### Geometric parameters (Å, °)

**Table:**

Fe1—C1	2.0269 (18)	C16—C17	1.399 (3)
Fe1—C7	2.030 (3)	C16—H16A	0.9500
Fe1—C8	2.033 (3)	C18—C19	1.398 (3)
Fe1—C2	2.044 (2)	C18—C23	1.405 (3)
Fe1—C6	2.048 (3)	C19—C20	1.380 (3)
Fe1—C5	2.049 (2)	C19—H19A	0.9500
Fe1—C3	2.051 (3)	C20—C21	1.391 (3)
Fe1—C9	2.053 (2)	C20—H20A	0.9500
Fe1—C4	2.055 (2)	C21—C22	1.382 (3)
Fe1—C10	2.057 (2)	C21—H21A	0.9500
P1—C24	1.837 (2)	C22—C23	1.391 (3)
P1—C18	1.839 (2)	C22—H22A	0.9500
P1—C17	1.843 (2)	C23—H23A	0.9500
P2—C5	1.818 (2)	C24—C25	1.387 (3)
P2—C36	1.842 (2)	C24—C29	1.393 (3)
P2—C30	1.844 (2)	C25—C26	1.388 (4)
O1—C11	1.437 (3)	C25—H25A	0.9500
O1—H1A	0.78 (3)	C26—C27	1.381 (4)
C1—C2	1.429 (3)	C26—H26A	0.9500
C1—C5	1.446 (3)	C27—C28	1.376 (4)
C1—C11	1.501 (3)	C27—H27A	0.9500
C2—C3	1.424 (3)	C28—C29	1.394 (3)
C2—H2A	0.9500	C28—H28A	0.9500
C3—C4	1.419 (3)	C29—H29A	0.9500
C3—H3A	0.9500	C30—C35	1.386 (3)
C4—C5	1.437 (3)	C30—C31	1.395 (3)
C4—H4A	0.9500	C31—C32	1.386 (3)
C6—C10	1.415 (4)	C31—H31A	0.9500
C6—C7	1.416 (5)	C32—C33	1.382 (4)
C6—H6A	0.9500	C32—H32A	0.9500
C7—C8	1.395 (5)	C33—C34	1.385 (3)
C7—H7A	0.9500	C33—H33A	0.9500
C8—C9	1.418 (4)	C34—C35	1.392 (3)
C8—H8A	0.9500	C34—H34A	0.9500
C9—C10	1.416 (4)	C35—H35A	0.9500
C9—H9A	0.9500	C36—C37	1.392 (3)
C10—H10A	0.9500	C36—C41	1.400 (3)
C11—C12	1.525 (3)	C37—C38	1.393 (3)
C11—H11A	1.0000	C37—H37A	0.9500
C12—C13	1.394 (3)	C38—C39	1.387 (3)
C12—C17	1.406 (3)	C38—H38A	0.9500
C13—C14	1.389 (3)	C39—C40	1.378 (4)
C13—H13A	0.9500	C39—H39A	0.9500
C14—C15	1.379 (3)	C40—C41	1.395 (3)
C14—H14A	0.9500	C40—H40A	0.9500
C15—C16	1.390 (3)	C41—H41A	0.9500
C15—H15A	0.9500		
			
C1—Fe1—C7	121.78 (11)	C8—C9—H9A	126.4
C1—Fe1—C8	158.24 (11)	Fe1—C9—H9A	126.2
C7—Fe1—C8	40.16 (14)	C6—C10—C9	108.3 (2)
C1—Fe1—C2	41.10 (8)	C6—C10—Fe1	69.50 (15)
C7—Fe1—C2	104.11 (11)	C9—C10—Fe1	69.69 (14)
C8—Fe1—C2	121.82 (11)	C6—C10—H10A	125.9
C1—Fe1—C6	106.09 (9)	C9—C10—H10A	125.9
C7—Fe1—C6	40.62 (13)	Fe1—C10—H10A	126.5
C8—Fe1—C6	67.92 (13)	O1—C11—C1	107.29 (16)
C2—Fe1—C6	118.70 (10)	O1—C11—C12	110.55 (16)
C1—Fe1—C5	41.54 (8)	C1—C11—C12	112.37 (16)
C7—Fe1—C5	160.76 (12)	O1—C11—H11A	108.9
C8—Fe1—C5	158.57 (12)	C1—C11—H11A	108.9
C2—Fe1—C5	69.20 (9)	C12—C11—H11A	108.9
C6—Fe1—C5	125.55 (10)	C13—C12—C17	119.70 (18)
C1—Fe1—C3	68.98 (9)	C13—C12—C11	118.09 (18)
C7—Fe1—C3	118.61 (12)	C17—C12—C11	122.20 (17)
C8—Fe1—C3	106.64 (12)	C14—C13—C12	121.0 (2)
C2—Fe1—C3	40.70 (10)	C14—C13—H13A	119.5
C6—Fe1—C3	153.77 (11)	C12—C13—H13A	119.5
C5—Fe1—C3	68.76 (9)	C15—C14—C13	119.7 (2)
C1—Fe1—C9	158.57 (10)	C15—C14—H14A	120.2
C7—Fe1—C9	68.04 (12)	C13—C14—H14A	120.2
C8—Fe1—C9	40.62 (12)	C14—C15—C16	119.85 (19)
C2—Fe1—C9	160.10 (10)	C14—C15—H15A	120.1
C6—Fe1—C9	68.02 (11)	C16—C15—H15A	120.1
C5—Fe1—C9	123.97 (10)	C15—C16—C17	121.5 (2)
C3—Fe1—C9	125.55 (11)	C15—C16—H16A	119.3
C1—Fe1—C4	69.23 (9)	C17—C16—H16A	119.3
C7—Fe1—C4	154.99 (12)	C16—C17—C12	118.18 (18)
C8—Fe1—C4	122.09 (12)	C16—C17—P1	121.89 (16)
C2—Fe1—C4	68.54 (10)	C12—C17—P1	119.88 (14)
C6—Fe1—C4	163.94 (10)	C19—C18—C23	118.26 (19)
C5—Fe1—C4	40.98 (8)	C19—C18—P1	117.21 (17)
C3—Fe1—C4	40.43 (9)	C23—C18—P1	124.51 (15)
C9—Fe1—C4	110.42 (11)	C20—C19—C18	121.0 (2)
C1—Fe1—C10	122.04 (9)	C20—C19—H19A	119.5
C7—Fe1—C10	67.90 (12)	C18—C19—H19A	119.5
C8—Fe1—C10	67.81 (11)	C19—C20—C21	120.3 (2)
C2—Fe1—C10	155.54 (10)	C19—C20—H20A	119.8
C6—Fe1—C10	40.32 (10)	C21—C20—H20A	119.8
C5—Fe1—C10	110.38 (9)	C22—C21—C20	119.6 (2)
C3—Fe1—C10	163.54 (10)	C22—C21—H21A	120.2
C9—Fe1—C10	40.29 (10)	C20—C21—H21A	120.2
C4—Fe1—C10	128.20 (10)	C21—C22—C23	120.4 (2)
C24—P1—C18	101.26 (10)	C21—C22—H22A	119.8
C24—P1—C17	102.60 (9)	C23—C22—H22A	119.8
C18—P1—C17	100.64 (9)	C22—C23—C18	120.43 (18)
C5—P2—C36	100.83 (9)	C22—C23—H23A	119.8
C5—P2—C30	98.80 (10)	C18—C23—H23A	119.8
C36—P2—C30	103.98 (9)	C25—C24—C29	118.6 (2)
C11—O1—H1A	110 (2)	C25—C24—P1	124.09 (18)
C2—C1—C5	107.90 (17)	C29—C24—P1	117.28 (18)
C2—C1—C11	126.3 (2)	C24—C25—C26	120.8 (3)
C5—C1—C11	125.77 (18)	C24—C25—H25A	119.6
C2—C1—Fe1	70.09 (11)	C26—C25—H25A	119.6
C5—C1—Fe1	70.06 (11)	C27—C26—C25	120.0 (3)
C11—C1—Fe1	125.88 (13)	C27—C26—H26A	120.0
C3—C2—C1	108.1 (2)	C25—C26—H26A	120.0
C3—C2—Fe1	69.92 (13)	C28—C27—C26	119.9 (3)
C1—C2—Fe1	68.82 (11)	C28—C27—H27A	120.0
C3—C2—H2A	126.0	C26—C27—H27A	120.0
C1—C2—H2A	126.0	C27—C28—C29	120.2 (3)
Fe1—C2—H2A	126.9	C27—C28—H28A	119.9
C4—C3—C2	108.5 (2)	C29—C28—H28A	119.9
C4—C3—Fe1	69.94 (15)	C24—C29—C28	120.4 (3)
C2—C3—Fe1	69.38 (14)	C24—C29—H29A	119.8
C4—C3—H3A	125.7	C28—C29—H29A	119.8
C2—C3—H3A	125.7	C35—C30—C31	118.5 (2)
Fe1—C3—H3A	126.5	C35—C30—P2	124.53 (16)
C3—C4—C5	108.4 (2)	C31—C30—P2	116.88 (17)
C3—C4—Fe1	69.63 (15)	C32—C31—C30	120.8 (2)
C5—C4—Fe1	69.30 (12)	C32—C31—H31A	119.6
C3—C4—H4A	125.8	C30—C31—H31A	119.6
C5—C4—H4A	125.8	C33—C32—C31	120.2 (2)
Fe1—C4—H4A	126.8	C33—C32—H32A	119.9
C4—C5—C1	107.12 (18)	C31—C32—H32A	119.9
C4—C5—P2	127.69 (17)	C32—C33—C34	119.5 (2)
C1—C5—P2	125.19 (15)	C32—C33—H33A	120.2
C4—C5—Fe1	69.72 (12)	C34—C33—H33A	120.2
C1—C5—Fe1	68.39 (11)	C33—C34—C35	120.2 (2)
P2—C5—Fe1	126.60 (11)	C33—C34—H34A	119.9
C10—C6—C7	107.5 (2)	C35—C34—H34A	119.9
C10—C6—Fe1	70.18 (14)	C30—C35—C34	120.7 (2)
C7—C6—Fe1	69.01 (16)	C30—C35—H35A	119.7
C10—C6—H6A	126.2	C34—C35—H35A	119.7
C7—C6—H6A	126.2	C37—C36—C41	118.68 (19)
Fe1—C6—H6A	126.1	C37—C36—P2	123.54 (16)
C8—C7—C6	108.4 (3)	C41—C36—P2	117.47 (16)
C8—C7—Fe1	70.03 (17)	C36—C37—C38	120.9 (2)
C6—C7—Fe1	70.37 (15)	C36—C37—H37A	119.6
C8—C7—H7A	125.8	C38—C37—H37A	119.6
C6—C7—H7A	125.8	C39—C38—C37	119.8 (2)
Fe1—C7—H7A	125.4	C39—C38—H38A	120.1
C7—C8—C9	108.6 (3)	C37—C38—H38A	120.1
C7—C8—Fe1	69.81 (15)	C40—C39—C38	120.0 (2)
C9—C8—Fe1	70.43 (15)	C40—C39—H39A	120.0
C7—C8—H8A	125.7	C38—C39—H39A	120.0
C9—C8—H8A	125.7	C39—C40—C41	120.4 (2)
Fe1—C8—H8A	125.6	C39—C40—H40A	119.8
C10—C9—C8	107.2 (3)	C41—C40—H40A	119.8
C10—C9—Fe1	70.02 (14)	C40—C41—C36	120.2 (2)
C8—C9—Fe1	68.95 (16)	C40—C41—H41A	119.9
C10—C9—H9A	126.4	C36—C41—H41A	119.9
			
C7—Fe1—C1—C2	74.09 (18)	C2—Fe1—C7—C8	−123.03 (18)
C8—Fe1—C1—C2	44.9 (3)	C6—Fe1—C7—C8	119.1 (3)
C6—Fe1—C1—C2	115.44 (15)	C5—Fe1—C7—C8	170.0 (3)
C5—Fe1—C1—C2	−118.63 (17)	C3—Fe1—C7—C8	−81.9 (2)
C3—Fe1—C1—C2	−37.34 (14)	C9—Fe1—C7—C8	37.71 (18)
C9—Fe1—C1—C2	−173.9 (3)	C4—Fe1—C7—C8	−53.8 (3)
C4—Fe1—C1—C2	−80.76 (14)	C10—Fe1—C7—C8	81.33 (19)
C10—Fe1—C1—C2	156.36 (14)	C1—Fe1—C7—C6	77.20 (18)
C7—Fe1—C1—C5	−167.29 (15)	C8—Fe1—C7—C6	−119.1 (3)
C8—Fe1—C1—C5	163.5 (3)	C2—Fe1—C7—C6	117.87 (16)
C2—Fe1—C1—C5	118.63 (17)	C5—Fe1—C7—C6	50.9 (4)
C6—Fe1—C1—C5	−125.93 (13)	C3—Fe1—C7—C6	158.99 (15)
C3—Fe1—C1—C5	81.28 (13)	C9—Fe1—C7—C6	−81.39 (17)
C9—Fe1—C1—C5	−55.3 (3)	C4—Fe1—C7—C6	−172.8 (2)
C4—Fe1—C1—C5	37.87 (12)	C10—Fe1—C7—C6	−37.76 (15)
C10—Fe1—C1—C5	−85.01 (14)	C6—C7—C8—C9	0.2 (3)
C7—Fe1—C1—C11	−47.0 (2)	Fe1—C7—C8—C9	−60.0 (2)
C8—Fe1—C1—C11	−76.2 (4)	C6—C7—C8—Fe1	60.16 (18)
C2—Fe1—C1—C11	−121.0 (2)	C1—Fe1—C8—C7	40.0 (4)
C6—Fe1—C1—C11	−5.6 (2)	C2—Fe1—C8—C7	73.1 (2)
C5—Fe1—C1—C11	120.3 (2)	C6—Fe1—C8—C7	−37.87 (19)
C3—Fe1—C1—C11	−158.4 (2)	C5—Fe1—C8—C7	−171.0 (3)
C9—Fe1—C1—C11	65.1 (4)	C3—Fe1—C8—C7	114.9 (2)
C4—Fe1—C1—C11	158.2 (2)	C9—Fe1—C8—C7	−119.4 (3)
C10—Fe1—C1—C11	35.3 (2)	C4—Fe1—C8—C7	156.26 (18)
C5—C1—C2—C3	−1.0 (2)	C10—Fe1—C8—C7	−81.6 (2)
C11—C1—C2—C3	179.57 (19)	C1—Fe1—C8—C9	159.4 (2)
Fe1—C1—C2—C3	59.08 (16)	C7—Fe1—C8—C9	119.4 (3)
C5—C1—C2—Fe1	−60.13 (13)	C2—Fe1—C8—C9	−167.49 (16)
C11—C1—C2—Fe1	120.49 (19)	C6—Fe1—C8—C9	81.5 (2)
C1—Fe1—C2—C3	−119.74 (19)	C5—Fe1—C8—C9	−51.6 (4)
C7—Fe1—C2—C3	117.71 (17)	C3—Fe1—C8—C9	−125.72 (19)
C8—Fe1—C2—C3	78.19 (19)	C4—Fe1—C8—C9	−84.3 (2)
C6—Fe1—C2—C3	158.71 (15)	C10—Fe1—C8—C9	37.84 (17)
C5—Fe1—C2—C3	−81.22 (15)	C7—C8—C9—C10	−0.2 (3)
C9—Fe1—C2—C3	53.7 (4)	Fe1—C8—C9—C10	−59.81 (17)
C4—Fe1—C2—C3	−37.14 (14)	C7—C8—C9—Fe1	59.61 (19)
C10—Fe1—C2—C3	−174.9 (2)	C1—Fe1—C9—C10	−40.6 (3)
C7—Fe1—C2—C1	−122.56 (16)	C7—Fe1—C9—C10	81.27 (19)
C8—Fe1—C2—C1	−162.07 (16)	C8—Fe1—C9—C10	118.6 (3)
C6—Fe1—C2—C1	−81.55 (16)	C2—Fe1—C9—C10	151.3 (3)
C5—Fe1—C2—C1	38.52 (12)	C6—Fe1—C9—C10	37.31 (16)
C3—Fe1—C2—C1	119.74 (19)	C5—Fe1—C9—C10	−81.64 (17)
C9—Fe1—C2—C1	173.5 (3)	C3—Fe1—C9—C10	−168.46 (15)
C4—Fe1—C2—C1	82.59 (14)	C4—Fe1—C9—C10	−125.54 (15)
C10—Fe1—C2—C1	−55.2 (3)	C1—Fe1—C9—C8	−159.1 (3)
C1—C2—C3—C4	0.7 (3)	C7—Fe1—C9—C8	−37.3 (2)
Fe1—C2—C3—C4	59.15 (18)	C2—Fe1—C9—C8	32.7 (4)
C1—C2—C3—Fe1	−58.40 (15)	C6—Fe1—C9—C8	−81.2 (2)
C1—Fe1—C3—C4	−82.25 (15)	C5—Fe1—C9—C8	159.80 (19)
C7—Fe1—C3—C4	162.08 (16)	C3—Fe1—C9—C8	73.0 (2)
C8—Fe1—C3—C4	120.29 (17)	C4—Fe1—C9—C8	115.9 (2)
C2—Fe1—C3—C4	−120.0 (2)	C10—Fe1—C9—C8	−118.6 (3)
C6—Fe1—C3—C4	−166.0 (2)	C7—C6—C10—C9	−0.1 (3)
C5—Fe1—C3—C4	−37.56 (14)	Fe1—C6—C10—C9	59.08 (16)
C9—Fe1—C3—C4	79.76 (18)	C7—C6—C10—Fe1	−59.14 (17)
C10—Fe1—C3—C4	52.6 (4)	C8—C9—C10—C6	0.2 (3)
C1—Fe1—C3—C2	37.70 (13)	Fe1—C9—C10—C6	−58.97 (16)
C7—Fe1—C3—C2	−77.97 (17)	C8—C9—C10—Fe1	59.13 (19)
C8—Fe1—C3—C2	−119.76 (16)	C1—Fe1—C10—C6	−76.58 (17)
C6—Fe1—C3—C2	−46.1 (3)	C7—Fe1—C10—C6	38.04 (17)
C5—Fe1—C3—C2	82.39 (14)	C8—Fe1—C10—C6	81.55 (19)
C9—Fe1—C3—C2	−160.29 (14)	C2—Fe1—C10—C6	−37.0 (3)
C4—Fe1—C3—C2	120.0 (2)	C5—Fe1—C10—C6	−121.39 (15)
C10—Fe1—C3—C2	172.5 (3)	C3—Fe1—C10—C6	154.8 (3)
C2—C3—C4—C5	−0.2 (3)	C9—Fe1—C10—C6	119.7 (2)
Fe1—C3—C4—C5	58.64 (16)	C4—Fe1—C10—C6	−164.29 (15)
C2—C3—C4—Fe1	−58.80 (18)	C1—Fe1—C10—C9	163.73 (15)
C1—Fe1—C4—C3	81.58 (16)	C7—Fe1—C10—C9	−81.66 (19)
C7—Fe1—C4—C3	−39.7 (3)	C8—Fe1—C10—C9	−38.14 (18)
C8—Fe1—C4—C3	−77.59 (19)	C2—Fe1—C10—C9	−156.7 (2)
C2—Fe1—C4—C3	37.38 (14)	C6—Fe1—C10—C9	−119.7 (2)
C6—Fe1—C4—C3	157.3 (3)	C5—Fe1—C10—C9	118.92 (16)
C5—Fe1—C4—C3	120.0 (2)	C3—Fe1—C10—C9	35.1 (4)
C9—Fe1—C4—C3	−121.32 (16)	C4—Fe1—C10—C9	76.01 (18)
C10—Fe1—C4—C3	−163.36 (15)	C2—C1—C11—O1	−25.1 (3)
C1—Fe1—C4—C5	−38.37 (12)	C5—C1—C11—O1	155.63 (18)
C7—Fe1—C4—C5	−159.7 (2)	Fe1—C1—C11—O1	65.5 (2)
C8—Fe1—C4—C5	162.46 (15)	C2—C1—C11—C12	96.6 (2)
C2—Fe1—C4—C5	−82.57 (14)	C5—C1—C11—C12	−82.7 (2)
C6—Fe1—C4—C5	37.4 (4)	Fe1—C1—C11—C12	−172.79 (14)
C3—Fe1—C4—C5	−120.0 (2)	O1—C11—C12—C13	68.2 (2)
C9—Fe1—C4—C5	118.72 (14)	C1—C11—C12—C13	−51.7 (3)
C10—Fe1—C4—C5	76.69 (16)	O1—C11—C12—C17	−110.6 (2)
C3—C4—C5—C1	−0.5 (3)	C1—C11—C12—C17	129.5 (2)
Fe1—C4—C5—C1	58.36 (13)	C17—C12—C13—C14	−1.1 (3)
C3—C4—C5—P2	179.97 (17)	C11—C12—C13—C14	−179.9 (2)
Fe1—C4—C5—P2	−121.18 (17)	C12—C13—C14—C15	−1.4 (4)
C3—C4—C5—Fe1	−58.85 (18)	C13—C14—C15—C16	1.8 (4)
C2—C1—C5—C4	0.9 (2)	C14—C15—C16—C17	0.1 (3)
C11—C1—C5—C4	−179.67 (18)	C15—C16—C17—C12	−2.5 (3)
Fe1—C1—C5—C4	−59.20 (14)	C15—C16—C17—P1	−179.75 (17)
C2—C1—C5—P2	−179.50 (16)	C13—C12—C17—C16	3.0 (3)
C11—C1—C5—P2	−0.1 (3)	C11—C12—C17—C16	−178.27 (18)
Fe1—C1—C5—P2	120.36 (16)	C13—C12—C17—P1	−179.75 (16)
C2—C1—C5—Fe1	60.14 (13)	C11—C12—C17—P1	−1.0 (3)
C11—C1—C5—Fe1	−120.47 (18)	C24—P1—C17—C16	−11.30 (19)
C36—P2—C5—C4	−98.5 (2)	C18—P1—C17—C16	−115.52 (17)
C30—P2—C5—C4	7.7 (2)	C24—P1—C17—C12	171.52 (16)
C36—P2—C5—C1	82.08 (18)	C18—P1—C17—C12	67.30 (17)
C30—P2—C5—C1	−171.75 (17)	C24—P1—C18—C19	95.15 (18)
C36—P2—C5—Fe1	169.93 (13)	C17—P1—C18—C19	−159.55 (17)
C30—P2—C5—Fe1	−83.90 (14)	C24—P1—C18—C23	−86.39 (19)
C1—Fe1—C5—C4	118.94 (17)	C17—P1—C18—C23	18.90 (19)
C7—Fe1—C5—C4	153.5 (3)	C23—C18—C19—C20	−0.6 (3)
C8—Fe1—C5—C4	−44.3 (3)	P1—C18—C19—C20	177.94 (18)
C2—Fe1—C5—C4	80.82 (14)	C18—C19—C20—C21	−0.8 (4)
C6—Fe1—C5—C4	−168.09 (14)	C19—C20—C21—C22	1.3 (4)
C3—Fe1—C5—C4	37.08 (14)	C20—C21—C22—C23	−0.5 (4)
C9—Fe1—C5—C4	−82.29 (16)	C21—C22—C23—C18	−1.0 (3)
C10—Fe1—C5—C4	−125.33 (14)	C19—C18—C23—C22	1.5 (3)
C7—Fe1—C5—C1	34.6 (4)	P1—C18—C23—C22	−176.96 (16)
C8—Fe1—C5—C1	−163.3 (3)	C18—P1—C24—C25	23.5 (2)
C2—Fe1—C5—C1	−38.12 (12)	C17—P1—C24—C25	−80.3 (2)
C6—Fe1—C5—C1	72.98 (15)	C18—P1—C24—C29	−154.66 (17)
C3—Fe1—C5—C1	−81.86 (13)	C17—P1—C24—C29	101.60 (18)
C9—Fe1—C5—C1	158.78 (13)	C29—C24—C25—C26	0.4 (4)
C4—Fe1—C5—C1	−118.94 (17)	P1—C24—C25—C26	−177.7 (2)
C10—Fe1—C5—C1	115.73 (13)	C24—C25—C26—C27	−0.6 (4)
C1—Fe1—C5—P2	−118.55 (19)	C25—C26—C27—C28	−0.3 (4)
C7—Fe1—C5—P2	−84.0 (4)	C26—C27—C28—C29	1.4 (4)
C8—Fe1—C5—P2	78.2 (3)	C25—C24—C29—C28	0.7 (3)
C2—Fe1—C5—P2	−156.66 (18)	P1—C24—C29—C28	178.92 (19)
C6—Fe1—C5—P2	−45.57 (19)	C27—C28—C29—C24	−1.6 (4)
C3—Fe1—C5—P2	159.59 (17)	C5—P2—C30—C35	−87.0 (2)
C9—Fe1—C5—P2	40.23 (18)	C36—P2—C30—C35	16.6 (2)
C4—Fe1—C5—P2	122.5 (2)	C5—P2—C30—C31	89.93 (18)
C10—Fe1—C5—P2	−2.82 (17)	C36—P2—C30—C31	−166.51 (17)
C1—Fe1—C6—C10	120.89 (14)	C35—C30—C31—C32	2.1 (3)
C7—Fe1—C6—C10	−118.7 (2)	P2—C30—C31—C32	−174.96 (19)
C8—Fe1—C6—C10	−81.27 (17)	C30—C31—C32—C33	−1.4 (4)
C2—Fe1—C6—C10	163.48 (14)	C31—C32—C33—C34	0.1 (4)
C5—Fe1—C6—C10	79.59 (16)	C32—C33—C34—C35	0.4 (4)
C3—Fe1—C6—C10	−164.1 (2)	C31—C30—C35—C34	−1.6 (4)
C9—Fe1—C6—C10	−37.29 (15)	P2—C30—C35—C34	175.2 (2)
C4—Fe1—C6—C10	50.3 (4)	C33—C34—C35—C30	0.4 (4)
C1—Fe1—C6—C7	−120.38 (17)	C5—P2—C36—C37	14.0 (2)
C8—Fe1—C6—C7	37.46 (18)	C30—P2—C36—C37	−88.04 (19)
C2—Fe1—C6—C7	−77.79 (18)	C5—P2—C36—C41	−159.45 (16)
C5—Fe1—C6—C7	−161.68 (16)	C30—P2—C36—C41	98.54 (17)
C3—Fe1—C6—C7	−45.4 (3)	C41—C36—C37—C38	0.3 (3)
C9—Fe1—C6—C7	81.44 (18)	P2—C36—C37—C38	−173.05 (17)
C4—Fe1—C6—C7	169.0 (3)	C36—C37—C38—C39	−0.5 (3)
C10—Fe1—C6—C7	118.7 (2)	C37—C38—C39—C40	0.2 (3)
C10—C6—C7—C8	−0.1 (3)	C38—C39—C40—C41	0.3 (4)
Fe1—C6—C7—C8	−59.9 (2)	C39—C40—C41—C36	−0.6 (3)
C10—C6—C7—Fe1	59.88 (16)	C37—C36—C41—C40	0.3 (3)
C1—Fe1—C7—C8	−163.71 (16)	P2—C36—C41—C40	174.00 (17)

### Hydrogen-bond geometry (Å, °)

**Table:**

*D*—H···*A*	*D*—H	H···*A*	*D*···*A*	*D*—H···*A*
C11—H11A···P1	1.00	2.56	3.153 (2)	118
O1—H1A···C23	0.78 (3)	2.51 (3)	3.217 (3)	152 (2)
